# Choroideremia: The Endpoint Endgame

**DOI:** 10.3390/ijms241814354

**Published:** 2023-09-20

**Authors:** Maram E. A. Abdalla Elsayed, Laura J. Taylor, Amandeep S. Josan, M. Dominik Fischer, Robert E. MacLaren

**Affiliations:** 1 Oxford Eye Hospital, Oxford University Hospitals National Health Service Foundation Trust, Oxford OX3 9DU, UK; 2 Nuffield Laboratory of Ophthalmology, Nuffield Department of Clinical Neurosciences, University of Oxford, Oxford OX3 9DU, UK

**Keywords:** choroideremia, *CHM* gene, REP1, AAV, nonsense suppression therapy, antisense oligonucleotides, gene therapy, clinical trials, endpoints, outcome measures

## Abstract

Choroideremia is an X-linked retinal degeneration resulting from the progressive, centripetal loss of photoreceptors and choriocapillaris, secondary to the degeneration of the retinal pigment epithelium. Affected individuals present in late childhood or early teenage years with nyctalopia and progressive peripheral visual loss. Typically, by the fourth decade, the macula and fovea also degenerate, resulting in advanced sight loss. Currently, there are no approved treatments for this condition. Gene therapy offers the most promising therapeutic modality for halting or regressing functional loss. The aims of the current review are to highlight the lessons learnt from clinical trials in choroideremia, review endpoints, and propose a future strategy for clinical trials.

## 1. Introduction

### 1.1. Choroideremia and Genetics

Choroideremia is an X-linked retinal degeneration caused by the loss of function mutations involving the *CHM* gene, located on chromosome Xq21.2, which encodes the 95-kDa ubiquitously expressed Rab escort protein 1 (REP1) [1]. To date, over 500 unique variants have been reported in the *CHM* gene, and these tend to be null or loss-of-function variants (Leiden Open Variant Database). At least 76% of the *CHM*-reported alterations are gene/exon deletions, indels, and nonsense variants, while splice-sites and missense changes, respectively, represent the 16% and 6% [2]. REP1 is an enzyme that facilitates the post-translational prenylation of Rab GTPases, which are essential for the intracellular trafficking of vesicles, allowing them to dock with membranes, thereby facilitating the processes of phagocytosis, exocytosis, and intracellular transport. In the absence of functional REP1, unprenylated Rabs accumulate in the retinal pigment epithelial cells, leading to progressive dysfunction over many years and eventual apoptosis.

**Figure 1:**
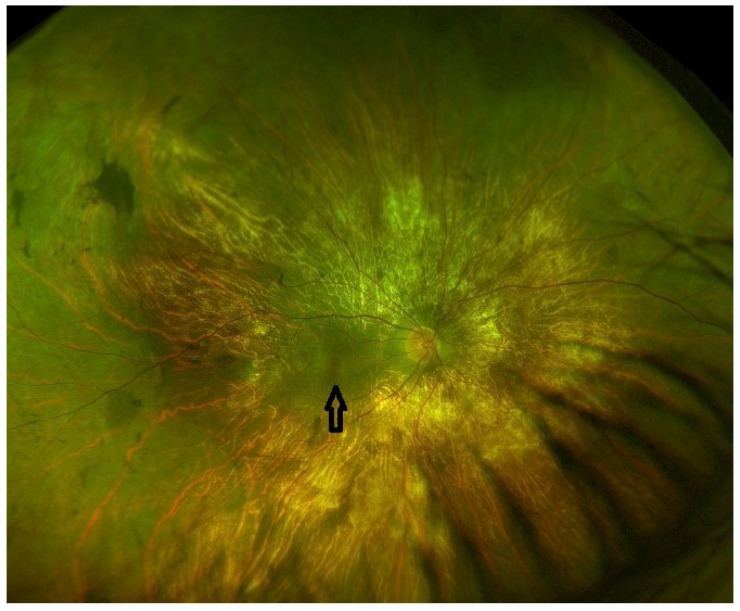
Pseudo-colour fundus image (Optos, Dumfernline, UK) demonstrating the choroideremia phenotype in an affected, non-treated male with no foveal involvement. The arrow points towards the residual island of preserved central macula.

**Figure 2:**
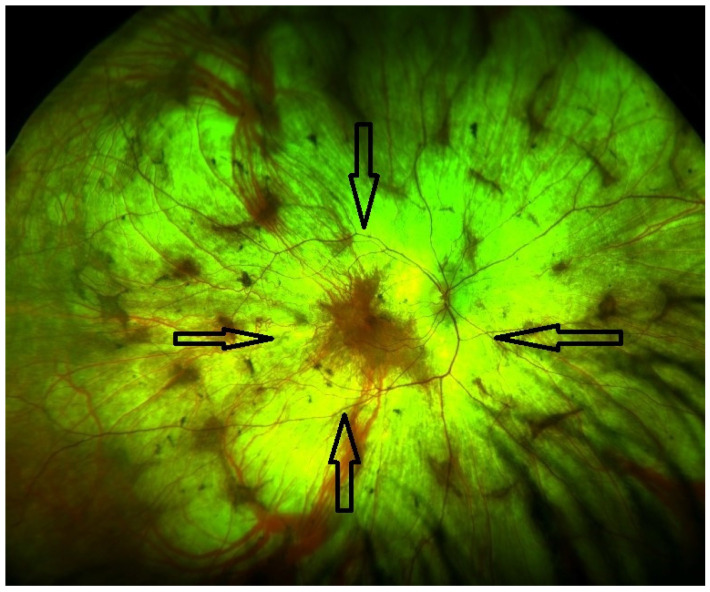
Pseudo-colour fundus image (Optos, Dumfernline, UK) demonstrating retinal degeneration advancement towards the fovea. There is extensive choroidal atrophy and the underlying sclera is visible.

**Figure 3:**
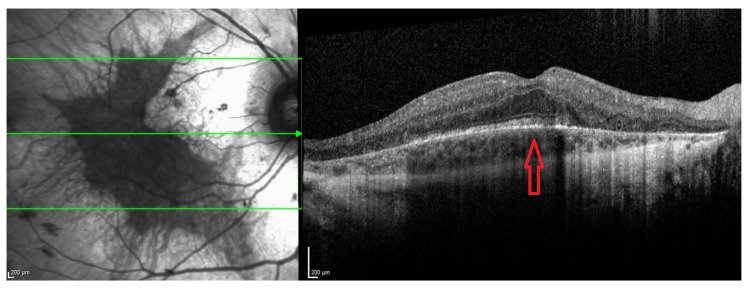
Spectral domain optical coherence tomography, Heidelberg, Germany shows outer retinal atrophy involving the fovea—the nasal macula is more severely affected than the temporal macula.

An autosomal homologue, the CHM-like gene (CHML, OMIM 118825), encodes Rab escort protein 2 (REP2) and is also ubiquitously expressed [3,4]. The Rab27a–REP1 complex appears to have a higher affinity for RabGGTaseII than for Rab27a-REP [5]. The mechanisms by which the under-prenylation of certain Rabs leads to retinal degeneration are not fully understood; however, it results in little variability in the choroideremia phenotype. Figure 1 demonstrates the fundal appearance of a non-treated choroideremia patient with no foveal involvement (the arrow points towards the intact fovea). Classically, there is progressive degeneration of the retina and choroid starting in the mid-periphery and extending both centripetally and anteriorly [6,7], as illustrated in Figure 2. Other genes involved in Rab prenylation do not appear to play any modifying role in the rate of disease progression [8]. In advanced disease, the fovea becomes involved (Figure 3).

More recently, it has been postulated that choroideremia may have potential extraocular manifestations related to oxidative stress and the metabolism of cholesterol, steroids, methylxanthines, fatty acids, drugs, and, notably, tryptophan [9]. Nevertheless, the effect of REP1 deficiency is mostly seen in retinal pigment epithelium and photoreceptor cells. Two hypotheses have been postulated to explain this bias in the spatial expression of this ubiquitous protein. First, retinal pigment epithelium and photoreceptor cells may happen to be more dependent on the delicate regulation of intracellular membrane trafficking, especially in light of the fact that these cells have limited regenerative potential, resulting in accelerated degeneration and apoptosis. A second hypothesis that has been proposed is that REP1 may have a selective affinity to particular Rabs that are more important for ocular cells than extraocular ones, as in the case of Rab27a and melanosome trafficking in retinal pigment epithelial cells [10] and in Rab6, 8 and 11 in photoreceptor outer segments [11].

### 1.2. Alternative Subretinal Gene Therapy Approaches for Choroideremia

#### 1.2.1. Nonsense Suppression Therapy

Whilst gene therapy clinical trials are ongoing, alternative therapies are also under investigation. These include nonsense suppression and antisense oligonucleotides.

Approximately 30% of *CHM* mutations are nonsense mutations resulting in a stop codon [12]; hence, nonsense suppression therapy may be a viable option. Translational readthrough-inducing drugs bind to the ribosomal subunit, increasing the ability of a near-cognate tRNA to compete with eukaryotic release factors for binding. An amino acid is then added to the peptide chain, resulting in the formation of a functional protein [13]. Despite the success of PTC124 in preclinical studies for restoring REP1 function in the chmru848 zebrafish model, this was not seen in human patient cells [14]. Although nonsense suppression therapy may be theoretically attractive, as it can be delivered systemically and non-invasively, efficacy is restricted by nonsense-mediated decay.

#### 1.2.2. Antisense Oligonucleotides

Antisense oligonucleotides have shown promise in both in vitro and in vivo models of RPE65 mutation-associated retinal dystrophy [15]. Small RNA sequences bind to cryptic splice sites in pre-mRNA, preventing splicing factors from binding and allowing a full-length REP1 gene product to form. Garanto et al. yielded an antisense oligonucleotide for choroideremia targeting the intronic splice mutation, c.315-4587 T>A, which resulted in the introduction of a 98-base-pair pseudo-exon and a stop codon [16]. In two patients, antisense oligonucleotide treatment did not restore REP1 expression in lymphoblast cells [16]. More recently, Zhai et al. showed the recovery of 83.2–95.0% of the normal RNA and 57.5% of the normal protein in fibroblasts from two trial patients with the c.1245-521A>G mutation using a customized 25-mer antisense oligonucleotide [17]. Since antisense oligonucleotide therapy is mutation-dependent, and a common splice variant has not been reported for *CHM*, this may not be as widely applicable as other therapies described. The recent trial of antisense oligonucleotide treatment with Sepofarsen for RPE65 mutation-associated retinal dystrophy has demonstrated risks such as cataractogenesis, retinal thinning, and cystoid macular changes [18]. It has to be appreciated that treatment with antisense oligonucleotides will be challenging, even if effective, as multiple administrations will be required over many decades, and injection-related complications are to be expected.

## 2. Lessons from Choroideremia Clinical Trials

Choroideremia lends itself to interventional therapeutic clinical trials (Table 1) because the phenotype can be widely recognized even in centers without genetic testing, and there is currently no cure [19]. Clinical trials of *CHM* gene therapy have also provided us with vital insights into how to obtain and evaluate outcome measures of clinical trials in conditions that are slowly progressive and in which visual acuity is maintained until late stages.

Much has been learnt about retinal surgical techniques, and instruments continue to be refined to minimize trauma and avoid complications and triggers of immunity [21,27]. An OCT-guided automated system for subretinal injection was developed to help identify the correct the plane of the subretinal space [28]. Robot-assisted infusion of the vector is also being developed to improve surgical consistency and safety [29].

The experience of the Oxford group has demonstrated the sustainability of the current *CHM* gene replacement protocols. Xue et al. demonstrated an improvement in visual acuity over the two-year trial period [28]. Longer-term follow-up with a mean of 3.6 years for the 12 protocol-treated participants confirmed that visual acuity gains were sustained [28]. Further insight was gained by the inclusion of the translational enhancer, Woodchuck hepatitis virus post-transcriptional regulatory element (WPRE), which could potentially increase transgene expression when compared with other Adeno-associated virus (AAV) constructs [30,31]. 

As per the experience of the Oxford group, data from other centers have also demonstrated good safety profiles for both the product and the technique of sub-macular vector delivery. The surgical techniques of the intravitreal or subretinal delivery of oligonucleotides or an AAV vector (required to deliver an RNA editing construct) [21] and its safety and lack of immunogenicity have been well established [32,33]. The subretinal approach, requiring a surgical component, is the gold standard at the present time and maintains strong momentum. Through the use of adaptive optics, Morgan et al. have recently demonstrated that the cone photoreceptor mosaic resettled on the retinal pigment epithelium following the resolution of the subretinal bleb at one month post-injection, remaining intact in eight of nine study participants, without widespread cone loss across the retinal area targeted by the retinal detachment [34]. This finding suggests that cone photoreceptors do not drop out as a consequence of mechanical or acute inflammatory changes in response to subretinal AAV2-hCHM [34].

Studying any rare disease remains a challenge. In the case of choroideremia, the patient pool is small and further restricted by rigid inclusion and exclusion criteria. Finally, sensitive, non-invasive biomarkers and clinical outcome measures for monitoring treatment responses are limited.

## 3. Clinical Endpoints

Longitudinal natural history studies have helped characterize the rate of disease progression in patients with choroideremia, aided our understanding of factors which predict the rate of progression and of how the phenotypes of affected males and females differ between and within these cohorts [7,35]. The relationships established have provided insight into the condition and will further guide the selection of outcome measures for choroideremia clinical trials.

### 3.1. What Makes a Good Outcome Measure

An outcome measure is a parameter that is used to gauge the effect of an intervention. An ideal outcome measure should be one that the treatment might plausibly affect, be indicative of an improved or worsened clinical outcome for the patient, and be a measure that can be compared across treatment groups with statistical accuracy. The larger the effect of the measure, the greater the statistical certainty of the interventional effect and/or the smaller the required sample size. This has traditionally been of limited concern in trials involving glaucoma or age-related macular degeneration due to the frequency of these ocular diseases and, hence, the large pool of potential recruits. In inherited retinal conditions such as choroideremia, however, recruiting a sufficient number of patients to gain statistical significance and gain confidence that a treatment effect exists can be extremely challenging. Here, a sensitive outcome measure could prove to be pivotal to clinical trial success.

In addition to measurable treatment effects, clinically meaningful, sensitive, and reproducible outcome measures for the intended population are paramount. For example, whilst diabetic retinopathy and retinal degenerations may both affect the retina, ocular coherence tomography parameters such as retinal thickness, used as outcome measures for diabetic retinopathy, would be insensitive and unsuitable as outcome measures in retinal dystrophies. As choroideremia affects vision, it would be intuitive for some aspect of visual function to serve as a primary outcome measure in clinical trials. When multiple aspects of visual function are used, e.g., from a comprehensive ocular assessment, then the primary outcome measure must be made clear at the start of the trial; otherwise, the interpretation of the trial results will be hindered by disparities between multiple outcome measures.

Best corrected visual acuity (BCVA) has traditionally been used as a primary gold standard outcome measure, but without clear justification in choroideremia trials. Should this concept be reformed and should outcomes be selected by expert consensus? A case in point is the use of lamotrigine, which separated from placebo on most secondary outcome measures but not on the primary outcome measure in the first trial in bipolar depression [36]; a subsequent meta-analysis of randomized controlled trials showed that the drug has a beneficial effect [37].

#### 3.1.1. Target Population

The target population for trials in choroideremia needs to be carefully considered. For stage I trials, patients with advanced disease are typically enrolled to primarily establish safety. It may be that intervention for halting disease progression or potential functional gains is more likely to be observed in patients with earlier disease, but this group of patients is excluded from such studies. In patients with advanced disease, an escalated dose may be required to give a detectable efficacy outcome. However, it would be unethical to use high doses of the interventional drug without first evaluating the safety and efficacy of lower doses. It may also be that the lower doses of the drug are indeed efficacious but due to limited areas with the potential for rescue, effects may remain undetected in patients with advanced disease. The recruitment of patients with early choroideremia, with near normal vision, may have the most to gain from potential treatments but have significant ethical constraints due to the potential risks associated with a surgical intervention. In addition, without an extremely sensitive measure which is able to detect changes in early-stage disease patients, an unfeasibly long clinical trial follow-up may be required to demonstrate treatment efficacy.

#### 3.1.2. Length of Clinical Trials

The length of existing choroideremia trials is five years or less [20,21,22,23,24,25,26]. As the aim of the treatment is to halt further degeneration and the natural history of the condition is one of very slow decline, one may argue that patients need to be followed up for ten years or more to exhibit a meaningful outcome [38]. Shen et al.’s data reporting that BCVA in eyes with choroideremia follows a two-phase linear decline with a transition age of approximately 39 years suggest that disease biomarkers that are measurable before this age may need to be taken into account when enrolling young patients [39].

#### 3.1.3. Choice of Controls

Though challenging, the appropriate use of controls in trials of choroideremia is a necessary prerequisite. Regulators, such as the European Medicines Agency and US Food and Drug Administration, should consider trial designs with contralateral eyes serving as the control when the disease is symmetrical, as the fellow eye would be expected to behave similarly. As most inherited retinal diseases show good or even excellent intra-individual symmetry, the use of fellow eye data as the control group might be a way to reduce noise in the dataset [6,40]. This would require:

(i) a demonstration that the inter-individual variability is greater than the intra-individual variability for the respective endpoint;
(ii) evidence that unilateral treatment has no effect on the endpoint measured in the fellow eye.

The use of both eyes is also an efficient use of data. However, the use of the fellow eye may introduce inherent statistical biases requiring multilevel statistical methods to account for nested data when determining whether outcome measures reach statistical significance [41]. Treating only one eye in a trial of a patient who would typically need both eyes treated in a clinical scenario raises difficulties in the economic analysis of the drug. The translation of functional improvement in a clinical trial with one eye treated to a clinical scenario with both eyes treated (and patients relying on the better eye for daily activity) is not straightforward: Food and Drug Administration endpoints generally rely on binocular functional vision.

### 3.2. Functional and Structural Outcome Measures Used in Previous Choroideremia Clinical Trials

Here, we summarize the various possible structural and functional outcome measures and their suitability for use in choroideremia clinical trials. For a rare disease such as choroideremia, it is prudent to consider both subjective and objective endpoints and to include the possibility of both conventional and novel measures. Observations from choroideremia trials inform the selection and ranking of endpoints.

### 3.3. Functional Outcome Measures

#### 3.3.1. Best Corrected Visual Acuity

Best corrected visual acuity (BCVA) using an Early Treatment Diabetic Retinopathy Treatment Study (ETDRS) chart and protocol is traditionally the leading outcome measure in many ophthalmic clinical trials [42,43]. It has remained popular due to its ease of use and cost-effectiveness. It is readily understood by examiners and patients and enables a simple interpretation of results.

BCVA has been the primary endpoint for all clinical trials of choroideremia to date. For FDA regulatory approval, a three-line (15 letter) gain is required to be considered a clinically significant treatment effect. However, in choroideremia, BCVA is preserved until late disease stages, due to the preservation of the central macular region. Therefore, in early-to-moderate disease stages, BCVA is within “normal limits”, making a gain in BCVA unlikely following gene therapy. Hagag et al.’s recent study showed that BCVA did not change in patients with choroideremia over the 12-month period [44]. BCVA is only impaired, in late disease stages, once degeneration encroaches into the central macular and foveal region. Impaired BCVA may provide a scope for meaningful improvement; however, it is also unknown whether these patients have sufficient areas of recoverable retina remaining to effect a rescue or enable a measurable treatment effect [45].

Overall, BCVA measures in choroideremia suffer from both large ceiling and floor effects, and the window of opportunity to measure a meaningful change due to novel treatment effects is extremely narrow. The insensitivity of BCVA to disease change is a widespread problem in other ocular and retinal diseases, and initiatives are in place to explore alternative visual function assessments [46,47]. Moreover, the use of the ETDRS chart in patients with asymmetrically constricted visual fields may be a suboptimal strategy in choroideremia due to the greater temporal preservation of the retina [7]. This results in difficulties in reading the right compared to the left side of the EDTRS chart with the right eye [48]. Alternative visual function measures that can be used as endpoints must be considered for choroideremia.

#### 3.3.2. Low-Luminance Visual Acuity

Low-luminance VA (LLVA) involves the same ETDRS VA protocol but a 2.0 neutral density lens is placed in front of the viewing eye [49]. A study comparing the clinical utility of LLVA and low-contrast VA in choroideremia found that low-contrast VA (using 1.25% and 2.5% contrast ETDRS charts) was prone to floor and ceiling effects, whereas LLVA showed a greater dispersion of results across the spectrum of disease [50]. A further study showed that LLVA is less affected in early stages of the disease but appeared to be impaired prior to BCVA; therefore, it was concluded that LLVA loss is a marker of disease progression into advanced stages [51]. Low-luminance deficit is the difference between BCVA and LLVA, although the relative difference is useful to understanding the visual function around the foveal or preferred retinal locus; using the low-luminance deficit as an outcome measure should be approached with caution, as it is prone to additive variability from BCVA and LLVA testing [50].

#### 3.3.3. Microperimetry

Microperimetry enables an accurate assessment of central retinal sensitivity (also known as fundus-tracked perimetry); it uses the principles of static automated perimetry, combined with a real-time fundus image via a scanning laser ophthalmoscope. This live image is used by the device to automatically adjust the position of stimuli to compensate for small eye movements due to fixation losses. The result is a measure of localized retinal sensitivity values, which can be consistently measured over time to accurately assess changes to sensitivity in specific locations [52]. This makes the device ideal for use in clinical trials where subtle off-center therapeutic effects may otherwise go un-noticed or be attributed to natural variability. As such, the use of microperimetry in research and clinical trials has increased dramatically across the world, involving many ocular diseases, including in choroideremia. It is frequently indicated as either a secondary outcome measure in phase one trials or as a primary or secondary outcome measure in phase two or phase three trials.

Studies have shown that dark adaptation time and pharmacological pupil dilation are not necessary for microperimetry-testing patients with choroideremia [53,54]. Testing parameters such as grid selection need careful consideration; the rectilinear 10-2 grid has gained popularly, as it provided a large coverage across the central macular. However, it may be unsuitable in detecting sensitivity in later-stage patients with very small residual islands; here, the circular six-degree radial grid maybe better [6]. Sensitivity measures also need careful consideration. Individual pointwise sensitivity has been shown to be more variable (±8.4 dB), particularly in later-stage patients [6]. The standard sensitivity output is mean sensitivity; this is the average of all pointwise sensitivity points. Caution should be exercised in individuals with a large number of scotoma stimuli, particularly in patients with asymmetrical visual islands (a common characteristic in choroideremia), since non-seen points are arbitrarily assigned −1.0 dB, which significantly lowers the overall mean sensitivity. Volume sensitivity, based on the hill-of-vision principles, has been created as an alternative sensitivity measure for microperimetry. It is less affected by the averaging effects of non-seen points and is also less affected by the spatial weighting of radial grids [55].

#### 3.3.4. Scotopic Microperimetry

Scotopic microperimetry is a modified version of mesopic microperimetry; it combines two-color perimetry with fundus-controlled perimetry performed in very low light conditions. The technique enables the spatially resolved mapping of central retinal sensitivity alongside the ability to distinguish between rod and cone photoreceptor sensitivities, using cyan (505 nm) and red (627 nm) stimuli. Scotopic microperimetry has the potential to detect retinal sensitivity changes in patients with early-stage choroideremia; however, its utility as an outcome measure in choroideremia has yet to be explored [56].

#### 3.3.5. Full-Field Perimetry

Peripheral visual field loss in choroideremia can be measured using both static automated and kinetic perimetry. Kinetic perimetry is useful for defining the borders of scotoma regions and providing an intuitive global assessment of functional versus non-functioning areas of the retina. Typically, patients with moderate-stage choroideremia have large mid-peripheral scotomas, with small intact central and far peripheral islands. As the disease further advances, degeneration progresses both centripetally inward and radially outward. An early symptom in choroideremia, nyctalopia, may be measurable using two-color dark-adapted perimetry [57]; however, all forms of static perimetry suffer from a high test variability, which may obscure the treatment effects of novel gene therapies. In addition, a great deal of time is spent assessing what patients cannot see, rather then what they can, which, for patients, is very disheartening. For these reasons, clinical trial protocols have generally moved away from assessing full-field perimetry in patients with choroideremia. A modified-Esterman test, which provides a rapid suprathreshold binocular visual field assessment, has been recommended for use as a safety measure in clinical trials to monitor the longitudinal assessment of peripheral visual function, and it maybe also be useful for health economic analyses [58].

### 3.4. Full-Field Stimulus Testing (FST)

FST testing involves the assessment of chromatic sensitivity in different background lighting conditions, using the ganzfeld bowl stimulator, to assess the specific photoreceptor type. For a full developmental history, we refer the reader to a recent article detailing the origins of FST and its use in Leber congenital amaurosis trials [59]. The test was designed to assess sensitivity in very-low-vision patients who were unable to fixate sufficiently to perform standard or dark-adapted perimetry. It became a prominent outcome measure in *RPE65*-associted retinal dystrophy clinical trials [45]. Since FST provides a global response to stimuli with no spatial information, changes in global threshold levels using FST in early-to-moderate choroideremia disease (intact foveal function) is unlikely to be sufficiently sensitive to detect meaningful changes due to therapeutic effects. In addition, as choroideremia patients retain relatively good vision until late into the disease progression, the ability of FST to detect subtle changes is unlikely to be superior to BCVA testing or that of other psychophysical tests. In later-stage disease, where central vision is lost but peripheral islands remain, and fixation performance is insufficient to allow for reliable perimetry or microperimetry testing, FST may be useful in assessing therapeutic effects targeted for off-center islands of vision [45].

### 3.5. Patient-Reported Outcome Measures

A patient-reported outcome is “any report of the status of a patient’s health condition that comes directly from the patient, without interpretation of the patient’s response by a clinician or anyone else” [60]. They are highly recommended by the FDA to be incorporated into a clinical trial [61], although they are not needed for regulatory drug approval. Patient-reported outcomes are of value in clinical trials, as they offer a means for the systematic collection of patient perspectives.

There are a large range of patient-reported outcome measures assessing vision-related quality of life—some designed for specific conditions or patient populations, and others designed to assess specific areas of visual function such as low vision or mobility, while other questionnaires are more generic [61]. The National Eye Institute Visual Function Questionnaire-25 (VFQ-25) is the most widely used; it was developed to assess vision-related quality of life as a consequence of ocular disease [62] and has been used as an outcome measure in several Choroideremia gene therapy clinical trials [22,23]. The VFQ-25 has been shown to be reliable and valid in a range of eye diseases including cataracts, glaucoma, age-related macular degeneration and diabetic retinopathy as well as inherited retinal disease including retinitis pigmentosa [63,64,65,66,67]. A Japanese study reported a highly significant correlation between the vision-related quality of life score generated from the VFQ-25 and the extent of peripheral vision loss in patients with retinitis pigmentosa (r = 0.519, *p* = 0.0006) [68]. In choroideremia, the VFQ-25 was used in a longitudinal study; however, the results showed no significant differences in visual function and quality of life between those with early disease (group 1) and those with later disease stages (group 2) [44]. To the best of our knowledge, the utility and validation of the VFQ-25 or any other patient-reported outcome measure specifically for use in patients with choroideremia, remains unexplored [67,69,70]. A recent review of patient-reported outcome measures used in inherited retinal diseases reported that the VFQ-25 is unsuitable in this population as an outcome measure in gene therapy clinical trials and recommended the development of a novel patient report outcome measure instrument that meets the FDA guidelines for validity, reliability, ability to detect change, and interpretability [71].

Patient-reported outcome measures are important since patient quality of life is arguably the most important factor when evaluating treatment efficacy [61]. They also support health economic analysis for determining whether a study or new treatment is cost-efficient. However, in gene therapy clinical trials, it is convention to only treat one eye (typically the worst-seeing eye) and use the second eye as a control. Since patient-reported outcome measures typically represent visual function from the best-seeing eye [72], in preservation gene therapy, the patient-reported outcome measure is likely to have limited potential in measuring any significant therapeutic change.

#### Multi-Luminance Mobility Testing (MLMT)

With the mobility maze, SPARK has paved the way for novel functional visual outcomes to be considered in clinical trials of inherited retinal disease [73]. The mobility maze involves patients navigating through a maze under different ambient lighting conditions whilst they are assessed for walking accuracy and speed [73]. Although patients with choroideremia struggle with mobility due to a loss of peripheral vision, current therapeutic trials are focused on treating the macular region. As the current therapeutics do not target peripheral retinal function, MLMT is unlikely to be able to detect any marked therapeutic response in these individuals. One should also be cognizant of the significant expense and logistical challenges associated with implementing MLMT. Furthermore, variability has been noted as a significant issue with this endpoint, which requires further validation. 

### 3.6. Structural Outcome Measures

#### 3.6.1. Ocular Coherence Tomography (OCT)

The ellipsoid zone is thought to either arise due to the light scatter of the mitochondria within the ellipsoid region of the inner segments [74] or refractive changes between the inner/outer segment junction [75]. Whilst the extent of the ellipsoid zone may be highly correlated with that of the fundus autofluorescence region [76], it has been found that ellipsoid zone loss generally precedes that of fundus autofluorescence changes [77]. As such, ellipsoid zone loss may be a suitable clinical trial outcome measure in early stages of the disease [35]. However, this would be contingent on whether the burden of intensive manual image processing can be overcome. In later stages of the disease, the ellipsoid zone integrity may have deteriorated such that it cannot easily be resolved on ocular coherence tomography b-scans. Fundus autofluorescence changes may also slow during the late disease stage, making the accurate evaluation of changes due to treatment effects difficult. In such cases, visual acuity, microperimetry, mobility tests, and patient-reported outcomes may be more appropriate measures for revealing altered visual function.

#### 3.6.2. Fundus Autofluorescence

Fundal autofluorescence can demonstrate retinal pigment epithelium integrity, with autofluorescent intensity being an indicator of the lipofuscin content. Fundus autofluorescence may therefore be used as a sensitive measure in early disease [78]. Similarly, the ellipsoid zone area on ocular coherence tomography reveals the extent of the remaining photoreceptors and has been investigated as a potential structural marker and as a clinical trial endpoint [77]. The most potentially useful parameters for monitoring disease progression were parameters from ocular coherence tomography and fundal autofluorescence. Disease progression was inversely correlated with age (slower in patients 50 years of age or older). The study demonstrated the need for highly skilled photographers and graders due to high levels of inter- and intra-observer measurement variability [79]. Poli et al. recently demonstrated the correlation of three distinct retinal pigment epithelium patterns on fundal autofluorescence with microperimetry in choroideremia patterns [80]. This finding helps support the use of fundus autofluorescence as the primary endpoint in future choroideremia clinical trials in patients with early disease.

#### 3.6.3. Concluding Remarks about Endpoints

Despite the use of appropriate outcome measures being highlighted as an important factor in the success of ocular gene therapy trials, there is no coherent approach worldwide. It is worthwhile noting that other fields have highlighted similar issues for gene therapy. To capture meaningful outcomes, we propose utilising different outcome measures for patients of different disease stage (Table 2). As choroideremia is a rare disease, it is judicious to consider both subjective and objective endpoints. 

## 4. Future Directions for Choroideremia Clinical Trials

### 4.1. Earlier Intervention

Trials so far have recruited patients with moderate–advanced disease and have showed improved or sustained BCVA in a subgroup who would have been predicted to have rapid visual acuity loss. A meta-analysis of visual acuity in choroideremia revealed that BCVA as a function of age followed two phases: slow followed by rapid decline, with an estimated transition age of 39 years [39]. Potentially, earlier intervention in younger patients with larger areas of residual retina would carry a greater chance of maintaining the patient’s vision, and this needs to be addressed in future trials. Electrophysiological parameters for assessing a global macular response may be a more relevant outcome measure in this group.

### 4.2. Novel Structural and Functional Measure

Future structural and functional outcome measures may make use of adaptive optics [81] to increase the resolution from the current generation of scanning laser ophthalmoscopes to enable the direct viewing and assessment of the photoreceptor mosaic. The possibility of directly assessing retinal rescue through the photoreceptor cell count or the assessment of morphological changes would provide compelling evidence of a therapeutic effect but must be used in conjunction with functional measures and patient-reported outcomes to assess viability as a clinically significant treatment effect. Adaptive optics is still primarily a research tool, with results being difficult to interpret. In the near term, it is more likely that adaptive optics scanning laser ophthalmoscopes will be used as secondary or exploratory measures to help direct future trials and build upon evidence obtained from more traditional structural and functional measures. To date, these devices are not commercially available but are custom-built for individual research departments. Adaptive optics principles have also been applied to microperimetry [82] in order to place stimuli with greater precision. With the potential future adoption of adaptive optics technology, along with the vast increase in imaging data arising as a result, the concurrent development of artificial intelligence automated image analysis algorithms [83] is likely to occur, along with an increasing role for medical data scientists.

Virtual reality MLMT [84] has the potential to avoid many of the limitations associated with setting up a dedicated large space for MLMT. However, the virtual aspect of the tasks of navigating a maze may prove to be challenging to fully validate. The differences between navigating a virtual as opposed to a physical maze may be fundamental, with too many confounding factors to provide confidence that an improvement in one task translates well to an improvement in the other. A great deal of further research would be needed to establish a strong correlation between any virtual versus physical measures.

### 4.3. Optical Coherence Tomography Angiography

Another mode of retinal imaging worth consideration is optical coherence tomography angiography (OCT-A), as it is a non-invasive and easy-to-perform test. It has shown early and distinct changes in choroideremia, and this might, in the future, provide valuable insight into the degenerative process at an anatomical level. OCT-A may be more sensitive than standard techniques currently used such as fundus autofluorescence; it has shown profound alterations in the retinal vascular networks of choroideremia patients [85], and Arrigo et al. have recently demonstrated thinning of the inner nuclear layer and inner plexiform layers in the atrophic as well as in the apparently preserved retina [86]. Such changes may serve as valuable clinical endpoints worthy of consideration in early-stage disease.

### 4.4. Future Therapies

Since choroideremia results in a slow degeneration where vision is maintained until advanced stages, an alternative approach is to slow degeneration to extend the lifetime of a patient’s vision. As has been attempted in age-related macular degeneration, retinal pigment epithelium transplantation may be an option, as we know that this is the primary source of the disease.

Although gene-editing technologies are being developed and show great promise, it is not clear how they will offer an advantage over gene replacement therapy, as the same challenges with slow degeneration and difficult endpoints would apply. All choroideremia cases to date have been caused by a loss of function mutations, and the 1.9kB coding sequence makes it readily encodable by adeno-associated virus vectors while still allowing space for a choice of promoters and other regulatory elements [87].

Further research is necessary to establish the level of REP1 expression that is required to slow retinal degeneration. Fry et al. showed that levels of wildtype CHM mRNA at 1% those of normal can preserve the majority of the retinal structure and function [88]. Moreover, a single editing event may have a prolonged duration of the effect [89]. Gaining insights into these challenges will provide a better understanding of choroideremia and similar inherited retinal degenerations.

## 5. Conclusions

It is useful to consider what clinical outcome measures and endpoints have been used previously and worked well, such as those used in gene therapy treatment for *RPE65*-associated retinal dystrophy. However, caution should be exercised, as these two conditions have very different clinical presentations, with patients having different functional capabilities. *RPE65*-associated retinal dystrophy presents as an early onset retinal dystrophy, presenting before age five with severe visual impairment due to central atrophy [90]. Whereas in choroideremia, patients present in late childhood with nyctalopia and progressive visual field loss, in choroideremia, visual acuity is preserved until late disease stages, typically the fourth decade [6].

Gene therapy treatment is targeted at the preserved central retina, resulting in quite a significant shift in the benefit-to-risk ratio in choroideremia patients at the disease stages that are usually considered for initial clinical trials. Existing trials are more likely to produce comparatively minor changes in outcome measures and perhaps instead show a slowed progression of the disease based on structural and functional parameters.

The use of inappropriate endpoints in clinical trials leads to findings which lack validity and result in the suboptimal use of stakeholder time, costs, and resources. The careful selection of outcome measures tailored to measure specific disease clinical characters is required, as opposed to a battery of tests “that has been used before” for other ocular pathologies. It remains underdetermined whether outcome measure selection should be based on a comprehensive ocular assessment, looking at every aspect of visual function, or should be tailored to the disease under investigation and the severity. It is likely that different outcome measures are needed to measure different stages of the disease.

Significant investments of resources are necessary from patient advocacy groups, experts in regulatory affairs and choroideremia patients, as well as funders to re-evaluate the trial design for choroideremia. Date presented recently from the Phase III STAR study of timrepigene emparvovec (BIIB111/AAV2-REP1) identified three-line gains in visual acuity seen in patients receiving a choroideremia gene therapy vector, which were not seen in the control group. The number meeting three lines, however, did not reach statistical significance. In contrast, the number of patients gaining two lines of vision was statistically significant compared to the control group. Unfortunately, this did not reach the primary endpoint, and hence, there was no regulatory approval [91].

Innovative solutions are helping address the inherent challenges of conducting interventional studies in rare inherited retinal diseases. For example, a major point of discussion at an FDA-requested Advisory Committee Meeting for voretigene neparvovec-rzyl (U.S. FDA 2017c) was retinal thickness; as in the phase III trial, the requirement specified a thickness of 100 μm or greater [92]. The dialogue amongst the Advisory Committee focused on “real world” conditions, and participants advised flexibility. Labeling thus reads that “Patients must have viable retinal cells as determined by the treating physician(s)” (Luxturna 2017). Although retinal thickness is a surrogate marker of retinal viability, it is not an actual measure of viable cells. This vague guideline further reinforces how little we know about the effects of gene therapy in end-stage diseases. We should be cognizant of the fact that choroideremia clinical trials were designed to target such end-stage patients. These were patients at the “nadir” of a slowly progressing disease; this is analogous to the initial clinical trials investigating statin use for prolonging survival, which included patients with established coronary heart disease. These 4444 patients had angina pectoris or previous myocardial infarction, and the aim was to evaluate statin use regarding survival [93]. Similarly, in choroideremia trials, patients had relatively advanced diseases with established retinal degeneration, and hence, the aim was to delay further visual loss.

In conclusion, the valuable lessons learnt from existing trials of choroideremia will inevitably allow for better clinical trial design, more meaningful results, and improved gene therapy treatments for disease in the foreseeable future.

## Figures and Tables

**Table 1:** Summary of choroideremia gene therapy trials to date.

Clinicaltrials.gov Identifier and Study Start Date	Phase	Drug Design	Location	Vector	Study Status	Outcome	Reference
01461213 (October 2011)	I/II	r.AAV-REP1	University of Oxford, UK	Subretinal	Completed	3.5 years—Two patients with poor baseline BCVA gained 11 and 21 letters. Three patients with good baseline BCVA maintained BCVA. One patient had a surgical complication leading to a lower dose of the vector and had a decline in BCVA from 6 months to 3.5 years, likely due to degeneration in the fovea.	[20,21,22]
02341807 (January 2015)	I/II	AAV2-hCHM	University of Philadelphia, USA	Subretinal	Active, not recruiting	2 years—unchanged BCVA in 13/15. Acute foveal thinning in one patient. Macular hole in one patient.	
02077361 (April 2015)	I/II	r.AAV-REP1	University of Alberta, Edmonton, Alberta, Canada	Subretinal	Completed	2 years—BCVA change of −8 to >15 letters. One serious adverse event: a localized intraretinal immune response.	[23]
02553135 (September 2015)	II	r.AAV-REP1	University of Miami, Miami, USA	Subretinal	Completed	2 years—BCVA change of −1 to +10 letters.	[24]
02671539 (January 2016)	II	r.AAV-REP1	Tuebingen, Germany	Subretinal	Completed	2 years—mean change in BCVA of +3.7 letters.	[25,26]
02407678 (August 2016)	II	r.AAV-REP1	University College London & University of Oxford, UK	Subretinal	Completed	Awaited	
03507686 (November 2017)	II	r.AAV-REP1	Gemini, Biogen	Subretinal	Completed	Awaited	
03496012 (December 2017)	III	Low-dose and high-dose r.AAV-REP1	STAR, Biogen	Subretinal	Completed	Failed to meet primary and secondary endpoints.	
04483440 (June 2020)	1	AAV capsid variant (4D-100) carrying a transgene encoding a codon-optimized human *CHM* gene	4D Molecular Therapeutics	Intravitreal	Ongoing	Initial clinical safety data at both of the two dose levels in the 4D molecular therapeutics trial indicate that it is well tolerated and did not result in any dose-limiting toxicity (n = 6; all patients followed up for between one and nine months).	

**Table 2:** A summary of proposed outcome measures for different stages of disease.

Stage	Defining Characteristics	Potentially Appropriate Outcome Measures
Early stage	Rod degeneration in the mid-peripheral region with some secondary cone involvement and reduced para-central retinal sensitivity.	- Full field perimetry (static) - Mesopic/scotopic microperimetry - Patient-reported outcomes - OCT - FAF - Infrared AF
Moderate stage	Radial advancement of rod and secondary cone degeneration with a scotomatous mid-peripheral region and reduced retinal sensitivity encroaching the macula.	- Full field perimetry (static or kinetic) - Mesopic microperimetry - Patient-reported outcomes - OCT - FAF - BCVA/LLVA
Late stage	Defined by the encroachment of scotomas and/or reduced sensitivity well within the vascular arcades and encroaching the central region (Figure 1 and Figure 2).	- Full field perimetry (static or kinetic) - Mesopic microperimetry - Patient-reported outcomes - OCT (Figure 3) - FAF - FST - BCVA/LLVA

## Data Availability

Not applicable.
